# The dark side of AI in education: AI dependency as a mediator linking academic self-efficacy and teacher support to learning burnout among university students

**DOI:** 10.3389/fpsyg.2026.1889053

**Published:** 2026-09-10

**Authors:** Qian Huang, Shuang Tu, Jiayuan Lin, Li Lu, Chunlan Lv

**Affiliations:** 1 College of Management Science, Chengdu University of Technology, Chengdu, China; 2 School of Law (School of Intellectual Property), Xihua University, Chengdu, China; 3 College of Humanities and Management, Fujian University of Traditional Chinese Medicine, Fuzhou, China; 4 Tourism School, Sichuan University, Chengdu, China; 5 School of Economics and Management, Yibin University, Yibin, China

**Keywords:** academic self-efficacy, AI dependency, learning burnout, mediator, teacher support, university students

## Abstract

The rapid integration of AI technologies into higher education has reshaped students' learning experiences, yet also raised concerns regarding AI dependency and its psychological consequences. This study investigates the relationships among academic self-efficacy, teacher support, AI dependency, and learning burnout among university students, with a particular focus on the mediating role of AI dependency. A quantitative cross-sectional research design was adopted, and data were collected from university students through an online structured questionnaire survey. Statistical analyses were conducted to assess the reliability and validity of the measurement constructs and to examine the relationships among the variables. The results indicate that both academic self-efficacy and teacher support are negatively associated with learning burnout and are negatively associated with AI dependency. Furthermore, AI dependency is significantly and positively associated with higher levels of learning burnout. Mediation analysis reveals that AI dependency mediates the relationships between academic self-efficacy and learning burnout, as well as between teacher support and learning burnout. These findings deepen the understanding of the psychological processes underlying AI-assisted learning by identifying AI dependency as an important explanatory factor associated with the relationship between psychological and contextual factors and university students' learning burnout. The study also offers practical implications for educators, higher education institutions, and policymakers to promote responsible AI use and reduce learning burnout among university students.

## 1 Introduction

Artificial intelligence (AI) has rapidly transformed higher education by reshaping how students access information, acquire knowledge, and complete academic tasks (Hao et al., 2026; Wang et al., 2024; UNESCO, 2025). Unlike earlier education technologies such as e-learning platforms and teaching aids, AI-powered applications are widely accessible and increasingly used beyond institutional control due to their intuitiveness and versatility (OECD, 2026). AI-powered educational technologies, including intelligent tutoring systems, adaptive learning platforms, and generative AI applications, provide personalized learning experiences, real-time feedback, and flexible instructional support (Saaida, 2023; Nguyen and Nguyen, 2024). In particular, the emergence of large language model (LLM)-based tools such as ChatGPT, Gemini, and Copilot has significantly expanded students' access to AI-assisted learning (Luan et al., 2020). These technologies enable students to efficiently retrieve information, generate learning materials, solve problems, and improve academic productivity (Zhou, 2023; Srinivasa et al., 2022). Consequently, AI has become deeply integrated into student' everyday learning activities and is increasingly regarded as an important driver of learner-centered and technology-enhanced education.

However, the increasing use of AI tools has raised concerns regarding students' potential dependency on these technologies (King et al., 2023; Zhang et al., 2025). AI dependency refers to an excessive reliance on AI-assisted tools in completing academic tasks (Olawade et al., 2024). Although AI dependency falls within the broader domain of technology dependence and digital reliance, it represents a distinct form of reliance because the object of dependence shifts from technological functions and digital resources to AI-enabled cognitive capabilities. AI-powered systems can generate content, provide recommendations, and support problem solving beyond traditional technological support. Cognitive offloading focuses on the process of transferring cognitive tasks to external aids (Risko and Gilbert, 2016), whereas AI dependency reflects a sustained tendency to rely on AI for performing such tasks. In this sense, cognitive offloading may constitute one psychological mechanism underlying AI dependency. AI dependency should also be distinguished from several adjacent concepts. AI use generally refers to the utilization of AI tools, AI reliance denotes a functional tendency to seek AI assistance for task completion, whereas AI addiction describes compulsive AI use accompanied by significant functional impairment (Dagnino et al., 2026). Unlike these related concepts, AI dependency specifically captures excessive reliance on AI that may undermine autonomous learning. Such dependency may gradually develop because AI provides continuous cognitive support, personalized recommendations, and real-time feedback, reinforcing users' reliance on AI during learning (Xie et al., 2023; Yankouskaya et al., 2025). When AI reliance becomes excessive, AI dependency represents a transition in the role of technology, from serving as a learning aid to functioning as an external regulator of cognitive processes. AI dependency is treated as a psychological construct because it reflects individuals' cognitive evaluations, perceived necessity, and reduced autonomy. Accordingly, this study conceptualizes AI dependency as a comprehensive psychological phenomenon that encompasses emotional, cognitive, and behavioral dimensions, rather than being merely reflected in usage frequency (Goh et al., 2025). Although the term “dependency" is inherently neutral, this study specifically focuses on the maladaptive consequences arising from such dependency (Zhang et al., 2024).

Previous research has mainly focused on the developmental factors and behavioral consequences of AI dependency. For example, studies have shown that AI dependency may be reinforced by users' pursuit of efficiency, emotional attachment to AI systems, and AI characteristics such as privacy and nonjudgmental feedback (Yan et al., 2025; Kim et al., 2026; Al-Obaydi and Pikhart, 2026; Yin et al., 2026). Other studies have further revealed that excessive dependency on AI may weaken students' critical thinking ability, reduce active learning participation, and hinder the development of independent learning competence (Montag et al., 2020; Wijaya et al., 2024; Carobene et al., 2024; Tian and Zhang, 2025). These findings suggest that AI dependency extends beyond a simple pattern of technology use and may represent an important psychological concern in AI-supported learning environments. Among its potential psychological consequences, learning burnout has attracted increasing scholarly attention.

Among the psychological consequences of AI dependency, learning burnout has attracted increasing scholarly attention. Learning burnout is characterized by emotional exhaustion, cynicism, and diminished academic efficacy (Duru et al., 2014). In AI-powered learning environments, AI dependency may further intensify passive learning behaviors and diminish students' intrinsic motivation, thereby increasing the risk of burnout. Recent empirical evidence has begun to support this concern. For instance, Lan et al. employed the Interaction of Person-Affect-Cognition-Execution (I-PACE) model to explain how AI dependence contributes to learning burnout among university students (Lan et al., 2026). Wang et al. identified AI dependency as an important antecedent of burnout in technology-enhanced learning environments (Honggang et al., 2026). Similarly, Dong et al. found that inappropriate use of generative AI in programming courses may intensify students' learning burnout (Dong et al., 2025). Although emerging studies have identified AI dependency as a potential contributor to learning burnout, limited attention has been paid to the psychological and contextual factors that may influence AI dependency.

Although various educational factors may influence AI-supported learning, this study focuses on academic self-efficacy and teacher support as key individual and contextual resources that may shape students' AI dependency. Academic self-efficacy reflects students' beliefs in their ability to successfully accomplish academic tasks (Schunk, 2023). Students with lower self-efficacy are more likely to avoid challenging cognitive tasks and seek external assistance, thereby developing stronger reliance on AI tools over time (Yang et al., 2020; Uyar et al., 2026). Apart from academic self-efficacy, teacher support may also influence students' AI dependency. Teacher support, which includes emotional support, academic guidance, and constructive feedback, has long been recognized as a critical factor influencing students' academic engagement and psychological wellbeing (Lei et al., 2018). In AI-powered learning environments, teacher support may play an even more crucial role by guiding students' appropriate use of AI tools, becoming AI literate, and preventing over-dependency (National Education Association, 2024). Insufficient teacher support may increase students' dependency on AI systems as a substitute for instructional guidance, thereby exacerbating learning burnout. However, relatively little empirical research has examined how academic self-efficacy and teacher support may shape learning burnout through AI dependency in higher education.

To address these gaps, this study proposes a mediation model in which AI dependency serves as an intervening variable examining the associations between academic self-efficacy, teacher support, and learning burnout among university students. Specifically, students with higher academic self-efficacy and stronger teacher support may show lower levels of AI dependency, which may be associated with lower levels of learning burnout. By integrating psychological and contextual factors into a unified framework, this study seeks to provide a more comprehensive understanding of the factors associated with AI dependency and its relationships with learning outcomes in higher education. The main contributions can be summarized as follows. First, it extends the existing literature on AI in higher education by conceptualizing AI dependency as a key psychological factor that helps explain how psychological and contextual factors are associated with learning burnout. Second, it develops and empirically tests an integrated mediation model in which academic self-efficacy and teacher support influence learning burnout through AI dependency. Third, it enriches the understanding of learning burnout in emerging AI-driven learning environments by identifying AI dependency as a novel explanatory factor associated with learning burnout. Finally, the study offers practical implications for educators and institutions by highlighting the importance of enhancing students' academic self-efficacy and teacher support to reduce AI dependency and further alleviate learning burnout among university students.

The remainder of this paper is organized as follows. Section 2 presents the theoretical background and hypothesis development. Section 3 describes the research methodology. Section 4 reports the empirical results. Section 5 discusses the findings and their implications. Finally, Section 6 concludes the study.

## 2 Theoretical background and hypothesis development

### 2.1 Academic self-efficacy and AI dependency

Conservation of Resources Theory suggests that individuals strive to acquire, maintain, and protect valuable personal resources when coping with environmental demands (Hobfoll, 1989). From this perspective, academic self-efficacy represents an important psychological resource that enables students to effectively cope with academic challenges. Students with abundant psychological resources are less likely to seek compensatory external resources. Conversely, when students perceive insufficient self-efficacy, they may rely on AI-powered tools to compensate for perceived resource deficiencies. According to Albert Bandura's Social Cognitive Theory, academic self-efficacy reflects students' confidence in their ability to successfully complete academic tasks and solve learning problems independently (Bandura, 1986). Students with high academic self-efficacy tend to adopt autonomous learning strategies and rely more on their own cognitive abilities rather than external assistance, whereas students with low self-efficacy are more likely to seek technological support to compensate for perceived academic inadequacies. This explanation is further supported by Cognitive Offloading Theory, which suggests that individuals with lower confidence are more likely to delegate cognitively demanding tasks to external technologies, thereby increasing AI dependency (Sweller, 1988). Therefore, the following hypothesis is proposed:

**H1**: Academic self-efficacy is negatively associated with AI dependency.

### 2.2 Teacher support and AI dependency

From the perspective of Conservation of Resources Theory, teacher support constitutes an important contextual resource that provides academic guidance, emotional encouragement, and timely feedback. Such external resources enhance students' ability to cope with academic demands, thereby reducing their need to rely on AI-powered tools. Social Support Theory further suggests that adequate teacher support buffers academic stress and strengthens students' confidence in completing learning tasks independently (Cohen and Wills, 1985). Conversely, insufficient teacher support may increase students' reliance on AI as an alternative source of academic assistance. Therefore, teacher support may play a protective role by reducing students' reliance on AI. Accordingly, the following hypothesis is proposed:

**H2**: Teacher support is negatively associated with AI dependency.

### 2.3 AI dependency and learning burnout

Conservation of Resources Theory provides the primary explanation for the relationship between AI dependency and learning burnout. Although AI-powered tools may temporarily conserve cognitive resources by reducing learning effort, AI dependency may gradually weaken students' internal learning resources, such as critical thinking, problem-solving ability, and autonomous learning competence. As these psychological resources deteriorate, students become more vulnerable to emotional exhaustion and reduced academic accomplishment, ultimately resulting in learning burnout. Cognitive Offloading Theory further explains that habitual delegation of cognitively demanding tasks to AI reduces opportunities for active cognitive engagement, reinforcing this resource depletion process (Risko and Gilbert, 2016). Therefore, the following hypotheses are proposed:

**H3**: AI dependency is positively associated with learning burnout.

### 2.4 The mediating role of AI dependency

From the perspective of Conservation of Resources Theory, both academic self-efficacy and teacher support function as critical psychological and contextual resources that enable students to cope with academic stress and sustain learning engagement. Students with high academic self-efficacy tend to effectively regulate their learning behaviors and reduce the need to rely on AI-powered tools. Similarly, strong teacher support provides emotional reassurance, academic guidance, and feedback resources that buffer academic stress and discourage external cognitive substitution. In contrast, when students experience low academic self-efficacy or insufficient teacher support, they may perceive insufficiency of internal and external resources. To compensate for this resource loss, they are more likely to rely on AI tools as an alternative resource pool, thereby increasing AI dependency. Although such dependency may temporarily alleviate learning difficulties, it may undermine long-term cognitive development and academic engagement, ultimately intensifying learning burnout. Therefore, the following hypotheses are proposed:

**H4**: AI dependency mediates the relationship between academic self-efficacy and learning burnout.

**H5**: AI dependency mediates the relationship between teacher support and learning burnout.

**Figure 1:**
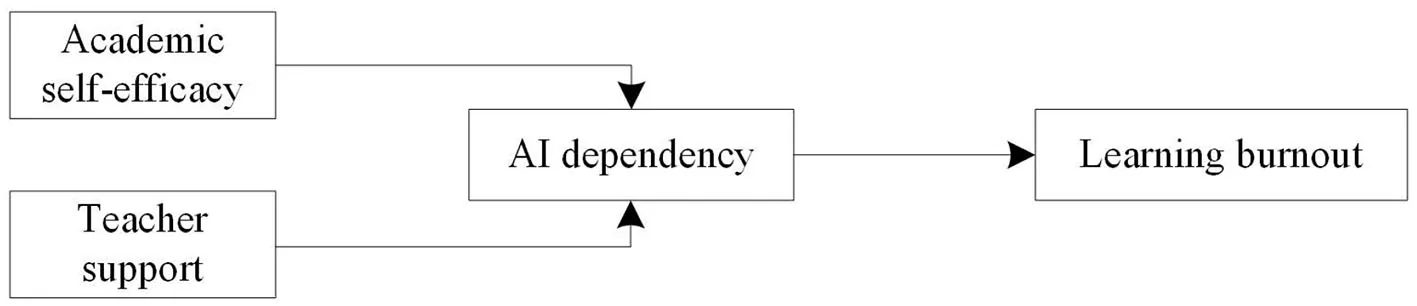
The conceptual model.

Based on the above hypotheses, the proposed conceptual model is presented in Figure 1.

## 3 Methods

### 3.1 Participants and procedures

This study adopted a cross-sectional research design to explore the associations among academic self-efficacy, teacher support, AI dependency, and learning burnout among university students, with AI dependency acting as a mediating variable in the conceptual model. An online structured questionnaire survey was conducted using the Questionnaire Star platform (www.wjx.cn), which is widely used for academic survey research in China (Deng and Yu, 2023). A convenience sampling approach was adopted, with participants recruited from undergraduate students enrolled in selected courses at several comprehensive universities in Chengdu, China. The questionnaire was distributed electronically during the data collection period, and participation was voluntary and anonymous. All participants were informed of the academic purpose of the study and the confidentiality policy. Attention check questions (e.g., “Please select strongly agree in this question.") were included in the questionnaire to ensure respondents paid sufficient attention (Muszyński, 2023). Data collection strictly followed ethical guidelines for behavioral and social science research.

A total of 311 questionnaires were distributed, of which 311 were returned. After excluding incomplete or invalid responses, 276 valid questionnaires were retained for subsequent analysis, yielding an effective response rate of 88.75%. Sample size requirements depend on the number of independent variables (Green and Samuel, 1991). In this study, the most complex regression model included three independent variables; therefore, the sample size of 276 was considered appropriate for the planned analyses. Moreover, the sample size was also adequate for measurement validation, as previous methodological research has suggested that samples exceeding 200 are generally acceptable for factor analysis (Comrey and Lee, 2013). In addition, the use of bootstrap procedures provides a reliable approach for testing indirect effects in mediation analysis without requiring normally distributed indirect effects (Hayes et al., 2013). A *post hoc* statistical power analysis was conducted using the *pwr* package in R. A multiple linear regression test for *R*^2^ deviation from zero was specified, with α = 0.05 and *N* = 276. The observed *R*^2^ values were converted to Cohen's *f*^2^ for each regression model. Specifically, the observed effect sizes were *f*^2^ = 0.220 for Model 1 (academic self-efficacy → AI dependency; *u* = 1), *f*^2^ = 0.049 for Model 2 (teacher support → AI dependency; *u* = 1), and *f*^2^ = 0.255 for Model 3 (AI dependency, academic self-efficacy, and teacher support → learning burnout; *u* = 3). The resulting statistical power values were >0.999, 0.967, and >0.999, respectively. All estimated power values exceeded the recommended criterion of 0.80, indicating adequate statistical power for the regression analyses underlying the proposed mediation model.

Among the 276 valid participants, 154 (55.80%) were male and 122 (44.2%) were female. In terms of academic level, freshmen accounted for 17.03%, sophomores 2.9%, juniors 66.3%, and seniors 13.77%, with juniors representing the largest proportion of the sample. This distribution reflected the enrollment composition of the selected courses, where junior students constituted the largest proportion of enrolled participants. Regarding AI learning tool usage frequency, 0.72% of participants reported rarely using AI tools (e.g., ChatGPT, DeepSeek, Doubao), while 15.58% used them 1–2 times per week, 27.54% used them 3–5 times per week, 23.55% used them 1–2 times per day, and 32.61% used them three or more times per day. For typical usage duration per session, 26.45% spent less than 15 min, 41.30% spent 15–30 min, 20.29% spent 30–60 min, and 11.96% spent more than 60 min.

### 3.2 Measures

The Academic Self-Efficacy Scale was developed by Owen and Froman (1988). In this study, the scale was used to assess students' confidence in their ability to independently manage learning tasks and maintain effective academic performance in AI-powered learning environments. Considering the widespread use of AI tools in education, several items were contextually modified to reflect students' perceptions of autonomous learning without excessive dependency on AI assistance. The scale consisted of eight items, including seven positively phrased items and one reverse-scored item. Sample items included “Even without using AI tools, I am confident that I can complete learning tasks independently” and “When learning tasks became more difficult, I was confident that I could cope through my own effort without excessively relying on AI tools.” The reverse-scored item assessed students' self-doubt when learning without AI assistance. All items were rated on a five-point Likert scale ranging from 1 (strongly disagree) to 5 (strongly agree). The Cronbach's alpha for the Academic Self-Efficacy Scale was 0.874.

The Teacher Support Scale was developed by Skinner and Belmont (1993). The teacher support was categorized into three dimensions: Structure (clear guidance, expectations, and assistance), Involvement (care, warmth, and understanding), and Autonomy Support (encouraging independent thinking, opinion expression, and providing choices). This multidimensional framework captured the extent to which teachers facilitated students' learning and engagement. Ten items were included: nine positively phrased items measuring three dimensions and one reverse-scored item. All items were rated on a five-point Likert scale from 1 (strongly disagree) to 5 (strongly agree). Cronbach's alpha value for the Teacher Support Scale was 0.915.

The AI Dependency Scale was adapted from Young's Internet Addiction Test (IAT) (Young, 1998), a widely used instrument for assessing technology-related dependency (Parmar and Kumbhakar, 2022). The IAT was selected as the conceptual basis because problematic reliance on Internet-related activities and AI-assisted tools share common psychological characteristics, including excessive reliance, difficulty reducing use, and perceived necessity. However, items were adapted to reflect AI-mediated academic activities rather than general Internet behaviors. Accordingly, rather than adopting all 20 original items, item selection was guided by two criteria: conceptual relevance to AI dependency and applicability to AI-assisted learning. Specifically, items related to excessive reliance on AI, compulsive engagement, and self-regulation difficulties were retained, whereas items referring to Internet-specific activities or consequences were excluded because they were not directly applicable to students' academic use of AI tools. The retained items were adapted to reflect students' academic use of AI tools while maintaining the original meaning of the items. For example, the item “Do you often find yourself spending more time online than expected?” was adapted to “I often use AI tools when completing coursework.” The final AI Dependency Scale consisted of seven items rated on a five-point Likert scale ranging from 1 (strongly disagree) to 5 (strongly agree). The Cronbach's alpha for the AI dependency Scale was 0.864.

The AI Dependency Scale was adapted from Young's Internet Addiction Test (IAT) (Young, 1998). Originally developed to assess problematic Internet use and dependence, the IAT has been widely used to examine technology-related dependency (Parmar and Kumbhakar, 2022). The IAT was selected as the conceptual basis because AI dependency shares key characteristics with problematic Internet dependence, particularly excessive reliance, difficulty controlling use, and perceived necessity. However, because the original IAT focuses on general Internet-related behaviors, it was adapted to reflect students' AI dependency in academic contexts rather than general Internet use. Accordingly, rather than adopting all 20 original items, the adaptation followed a two-stage process. First, items were screened according to their conceptual relevance to dependency and their applicability to AI-assisted learning. Items addressing excessive reliance, compulsive engagement, and difficulties in self-regulation were retained, whereas items referring specifically to Internet activities or Internet-related consequences were excluded because they were not directly applicable to academic AI dependency. Second, the retained items were reworded to refer specifically to students' use of AI tools for academic tasks while preserving the underlying meaning of the original items. For example, the item “Do you often find yourself spending more time online than expected?” was adapted to “I often find myself relying on AI tools more than intended when completing coursework.” The final AI Dependency Scale consisted of seven items rated on a five-point Likert scale ranging from 1 (strongly disagree) to 5 (strongly agree). The Cronbach's alpha for the AI Dependency Scale was 0.864.

The Learning Burnout Scale was derived from the Maslach Burnout Inventory-Student Survey (MBI-SS) developed by Schaufeli et al. (2002). The MBI-SS contained 15 items that assessed three dimensions including emotional exhaustion (five items), cynicism (six items), and diminished academic efficacy (four items) (López-Gómez et al., 2025). A shortened version of the scale was employed to improve measurement efficiency while maintaining reliability and validity. A total of nine items were selected to represent the three dimensions, with each dimension measured by three items. Sample items included: “I feel emotionally drained by my studies”, “I have become less enthusiastic about my studies”, and “I can effectively solve problems in my studies”. All items were rated on a five-point Likert scale, ranging from 1 (strongly disagree) to 5 (strongly agree), with higher scores indicating higher levels of learning burnout. The Cronbach's alpha for the Learning Burnout Scale was 0.934.

## 4 Results

All analyses were performed using SPSS 27. Regression analyses were conducted to test direct effects, and mediation analyses were performed using PROCESS macro Model 4. Standardized coefficients, confidence intervals, multicollinearity diagnostics, and model fit statistics were reported to improve statistical transparency.

### 4.1 Common method bias

Given that all variables were measured using self-reported questionnaires at a single time point, the potential influence of common method bias (CMB) was considered. Several procedural remedies were implemented, including ensuring anonymous responses and emphasizing that there were no right or wrong answers.

A full-collinearity VIF test was conducted to assess potential common method bias. Each of the four constructs was regressed on the other three constructs, and the resulting VIF values were examined. The maximum VIF values were 1.459 for AI Dependency, 1.579 for Academic Self-efficacy, 1.358 for Learning Burnout, and 1.365 for Teacher Support. All values were below the recommended threshold of 3.3, indicating no substantial common method bias based on the full-collinearity VIF assessment. A single-factor confirmatory factor analysis (CFA) was also conducted to examine whether the covariance among the observed items could be adequately explained by a single latent factor. The model demonstrated poor fit to the data (CFI = 0.372, RMSEA = 0.179), indicating that the observed covariance among the items could not be adequately explained by a single latent factor. Furthermore, an unmeasured latent method construct (ULMC) approach was attempted; however, the model resulted in a non-positive definite solution and was therefore not considered suitable for further interpretation. Harman's single-factor test was used as a supplementary diagnostic of potential common method variance (CMV). All measurement items were entered into an unrotated principal component factor analysis. The results revealed that the first unrotated factor accounted for 30.16% of the total variance.

Overall, the results provided no evidence of substantial common method bias in the present data.

### 4.2 Measurement model assessment

To validate the measurement structure of the study constructs, exploratory factor analysis (EFA) and confirmatory factor analysis (CFA) were conducted. Before factor extraction, the suitability of the data for factor analysis was examined. The results showed that the Kaiser–Meyer–Olkin (KMO) measure of sampling adequacy was 0.912, indicating excellent suitability for factor analysis. Bartlett's test of sphericity was also significant (χ^2^ = 7,521.004, df = 561, *p* < 0.001), confirming that the correlation matrix was appropriate for structure detection. An EFA was then performed using principal component analysis with varimax rotation. The results revealed four factors with eigenvalues greater than 1, which together explained 69.41% of the total variance. Subsequently, CFA was conducted to further assess the measurement model. The hypothesized four-factor model demonstrated an excellent fit to the data (χ^2^/df = 1.13, RMSEA = 0.04, CFI = 0.99, TLI = 0.98). The factor loadings of individual items are presented in Table 1.

**Table 1:** Factor loadings from CFA.

Item	Factor loading	Item	Factor loading
AI dependency1	0.787	Teacher support3	0.863
AI dependency2	0.850	Teacher support4	0.872
AI dependency3	0.819	Teacher support5	0.768
AI dependency4	0.752	Teacher support6	0.784
AI dependency5	0.788	Teacher support7	0.820
AI dependency6	0.793	Teacher support8	0.782
AI dependency7	0.679	Teacher support9	0.683
Academic self-efficacy1	0.855	Teacher support10	0.755
Academic self-efficacy2	0.850	Learning burnout1	0.645
Academic self-efficacy3	0.866	Learning burnout2	0.855
Academic self-efficacy4	0.802	Learning burnout3	0.830
Academic self-efficacy5	0.732	Learning burnout4	0.879
Academic self-efficacy6	0.690	Learning burnout5	0.803
Academic self-efficacy7	0.722	Learning burnout6	0.708
Academic self-efficacy8	0.640	Learning burnout7	0.746
Teacher support1	0.836	Learning burnout8	0.695
Teacher support2	0.882	Learning burnout9	0.750

The reliability and convergent validity of the measurement model were further evaluated using standardized factor loadings, Cronbach's alpha, Composite Reliability (CR), and Average Variance Extracted (AVE). As shown in Table 2, Cronbach's alpha coefficients ranged from 0.864 to 0.934, indicating satisfactory internal consistency. The CR values ranged from 0.874 to 0.935, exceeding the recommended threshold of 0.70, while the AVE values ranged from 0.538 to 0.618, all above the recommended threshold of 0.50. These results provide evidence of satisfactory reliability and convergent validity for all constructs, especially for the adapted AI Dependency Scale.

**Table 2:** Reliability and convergent validity.

Construct	Items	Cronbach's α	CR	AVE
AI dependency	7	0.864	0.874	0.538
Academic self-efficacy	8	0.874	0.891	0.546
Teacher support	10	0.915	0.915	0.618
Learning burnout	9	0.934	0.935	0.615

Discriminant validity was further evaluated using the Fornell–Larcker criterion and the Heterotrait–Monotrait ratio (HTMT). As shown in Table 3, the square roots of the AVE values for all constructs exceeded the corresponding inter-construct correlations, satisfying the Fornell–Larcker criterion. Furthermore, as presented in Table 3, all HTMT values ranged from 0.18 to 0.57, which were below the recommended threshold of 0.85 (Henseler et al., 2015), providing further evidence of satisfactory discriminant validity.

**Table 3:** Discriminant validity assessment.

Construct	1	2	3	4
**Panel A Fornell–Larcker criterion**				
1. AI dependency	**0.733**			
2. Academic self-efficacy	−0.424	**0.739**		
3. Teacher support	−0.217	0.514	**0.786**	
4. Learning burnout	0.451	−0.220	−0.170	**0.784**
**Panel B HTMT ratio**				
1. AI dependency	—			
2. Academic self-efficacy	0.48	—		
3. Teacher support	0.25	0.57	—	
4. Learning burnout	0.50	0.23	0.18	—

Bold diagonal values in Panel (A) represent the square roots of AVE; off-diagonal values represent Pearson correlation coefficients.

### 4.3 Descriptive statistics and correlations

Table 4 presents the means, standard deviations, and correlations among the study variables. AI dependency was negatively correlated with academic self-efficacy (*r* = −0.42, *p* < 0.01) and teacher support (*r* = −0.22, *p* < 0.01), and positively correlated with learning burnout (*r* = 0.45, *p* < 0.01). Academic self-efficacy was positively correlated with teacher support (*r* = 0.51, *p* < 0.01) and negatively related to learning burnout (*r* = −0.22, *p* < 0.01). Teacher support was also negatively associated with learning burnout (*r* = −0.17, *p* < 0.01). These results provide initial evidence supporting the hypothesized relationships.

**Table 4:** Descriptive statistics and correlation matrix.

Construct	Mean	SD	1	2	3	4
1. AI dependency	3.06	0.78	(0.86)			
2. Academic self-efficacy	3.49	0.69	−0.42^**^	(0.87)		
3. Teacher support	3.59	0.76	−0.22^**^	0.51^**^	(0.91)	
4. Learning burnout	2.83	0.89	0.45^**^	−0.22^**^	−0.17^**^	(0.93)

^**^p < 0.01 (two-tailed). Diagonal values represent Cronbach's alpha coefficients.

### 4.4 Hypothesis testing

Before interpreting the regression results, the regression assumptions were examined to ensure the appropriateness of the analyses. Three regression models were specified in this study: Model 1 examines the association between academic self-efficacy and AI dependency; Model 2 examines the association between teacher support and AI dependency; and Model 3 examines the association between AI dependency and learning burnout while controlling for academic self-efficacy and teacher support. As shown in Table 5, multicollinearity diagnostics indicated that the variance inflation factor (VIF) values ranged from 1.000 to 1.220, with tolerance values ranging from 0.820 to 1.000. These values met the recommended thresholds (VIF < 5 and tolerance > 0.10), suggesting that multicollinearity was not a concern. In addition, the Durbin–Watson statistics ranged from 1.981 to 2.091, which were close to 2 and indicated that the assumption of independent errors was satisfied.

**Table 5:** Regression assumption diagnostics.

Model	Tolerance	VIF	Durbin–Watson
Model 1	1.000	1.000	2.079
Model 2	1.000	1.000	2.091
Model 3	0.820–1.000	1.000–1.220	1.981

Regression analyses were conducted to examine the proposed direct relationships among variables, and the results are presented in Table 6. It can be seen that Academic self-efficacy was significantly and negatively associated with AI dependency (β = −0.424, *p* < 0.001, 95% CI [−0.603, −0.359]), explaining 18.0% of the variance in AI dependency (*R*^2^ = 0.180, *f*^2^ = 0.220). Thus, Hypothesis 1 was supported. Teacher support was also significantly and negatively associated with AI dependency (β = −0.217, *p* < 0.001, 95% CI [−0.337, −0.102]), accounting for 4.7% of the variance (*R*^2^ = 0.047, *f*^2^ = 0.049), supporting Hypothesis 2. Although academic self-efficacy and teacher support were associated with AI dependency, their explanatory power indicates that they account for only part of the variance in AI dependency. In particular, the relatively low *R*^2^ for teacher support indicates that it explains a relatively small proportion of the variance in AI dependency. Thus, the proposed model should be regarded as a partial rather than comprehensive explanation of AI dependency. Other individual, technological, and contextual factors, such as such as self-regulation, AI usage frequency, and learning environment, may also contribute to AI dependency. Theoretically, the proposed model may capture only part of the mechanisms underlying AI dependency, indicating that its explanatory scope is not exhaustive. Practically, the findings suggest that interventions targeting academic self-efficacy or teacher support alone may have limited effects on AI dependency and should be complemented by broader educational approaches.

**Table 6:** Regression estimates.

Variable	Model 1	Model 2	Model 3
Dependent variable	AI dependency	AI dependency	Learning burnout
Academic self-efficacy	−0.424^***^		
Teacher support		−0.217^***^	
AI dependency			0.451^***^
R^2^	0.180	0.047	0.203
f^2^	0.220	0.049	0.255

Standardized coefficients (β) are reported. ^***^p < 0.001.

Furthermore, AI dependency was significantly and positively associated with learning burnout (β = 0.451, *p* < 0.001, 95% CI [0.392, 0.634]), explaining 20.3% of the variance in learning burnout (*R*^2^ = 0.203, *f*^2^ = 0.255). Therefore, Hypothesis 3 was supported. According to Cohen's criteria, an *f*^2^ value of 0.02, 0.15, and 0.35 represents small, medium, and large effects, respectively. The effect size results suggest that academic self-efficacy and AI dependency had relatively stronger practical associations with the outcomes, whereas teacher support showed a smaller but meaningful contribution.

PROCESS Model 4 was selected to examine the indirect associations of academic self-efficacy and teacher support with learning burnout through AI dependency at the construct level. The measurement properties of the four constructs were first evaluated using EFA and CFA, which supported the hypothesized four-factor structure and demonstrated satisfactory reliability, convergent validity, discriminant validity, and model fit. Based on the validated measurement structure, composite scores were used to represent the four constructs in the subsequent mediation analysis. This construct-level approach was appropriate for the study objective of testing the hypothesized indirect associations. The mediation analysis used 5,000 bootstrap resamples to obtain 95% confidence intervals for the indirect effects. The mediation model is specified by the following regression equations:

$$\begin{array}{cc} M & =aX+e_{1}\\ Y & =cX+e_{2}\\ Y & =c^{\prime }X+bM+e_{3} \end{array}$$

where *X* represents academic self-efficacy or teacher support, *M* represents AI dependency, and *Y* represents learning burnout; *a* represents the association between *X* and *M*, *b* represents the association between *M* and *Y*, *c* represents the total association between *X* and *Y*, and *c*′ represents the direct association between *X* and *Y* after including *M*; *e*_1_, *e*_2_, and *e*_3_ represent residual error terms.

The bootstrap mediation results for the total, direct, and indirect effects are presented in Table 7. For Hypothesis 4, the indirect effect of academic self-efficacy on learning burnout through AI dependency was significant (indirect effect = −0.239, 95% CI [−0.331, −0.154]), as the bootstrap confidence interval did not include zero. The direct effect became non-significant after AI dependency was included as the mediator, suggesting a full mediation pattern. Therefore, Hypothesis 4 was supported. For Hypothesis 5, the indirect effect of teacher support on learning burnout through AI dependency was also significant (indirect effect = −0.143, 95% CI [−0.221, −0.071]), with the bootstrap confidence interval excluding zero. The direct effect became non-significant after AI dependency was included as the mediator, indicating a full mediation pattern. Therefore, Hypothesis 5 was supported.

**Table 7:** Bootstrap results for the mediation analysis.

Path	Total effect	Direct effect	Indirect effect	Boot SE	95% CI	PM
ASE → AID → LB	−0.284	−0.045	−0.239	0.052	[−0.331, −0.154]	84.2%
TS → AID → LB	−0.200	−0.057	−0.143	0.038	[−0.221, −0.071]	71.5%

ASE, academic self-efficacy; TS, teacher support; AID, AI dependency; LB, learning burnout. Bootstrap = 5,000 samples; CI, confidence interval; PM, proportion mediated (indirect effect / total effect × 100%).

**Figure 2:**
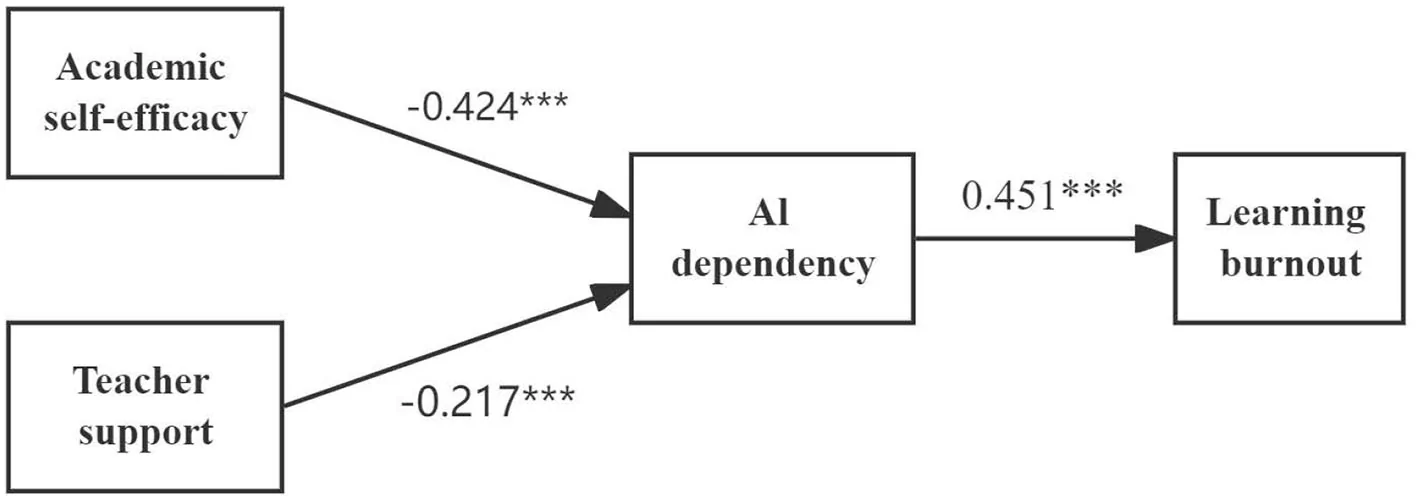
The mediation model of the relationships among academic self-efficacy, teacher support, AI dependency, and learning burnout. ***indicates *p* < 0.001.

Overall, the results supported all proposed hypotheses. AI dependency significantly mediated the associations between academic self-efficacy and learning burnout, as well as between- teacher support and learning burnout. The standardized regression results are summarized in Figure 2.

### 4.5 Alternative model analysis

To examine alternative explanations for the observed associations, reverse mediation models were additionally estimated. Although the reverse mediation models yielded statistically significant indirect effects, these effects were substantially smaller than those observed in the proposed model. Specifically, the indirect effect decreased from −0.239 (95% CI [−0.331, −0.154]) to −0.094 (95% CI [−0.163, −0.025]) for academic self-efficacy and from −0.143 (95% CI [−0.221, −0.071]) to −0.066 (95% CI [−0.128, −0.010]) for teacher support. These results are presented as supplementary evidence that is consistent with the proposed mediation model.

Partial mediation models were also estimated by adding direct paths from academic self-efficacy and teacher support to learning burnout while controlling for AI dependency. The direct effects of academic self-efficacy (*B* = −0.045, *SE* = 0.077, *p* = 0.559, 95% CI [−0.196, 0.106]) and teacher support (*B* = −0.089, *SE* = 0.065, *p* = 0.171, 95% CI [−0.217, 0.039]) on learning burnout were both non-significant after AI dependency was included. These findings are consistent with a full mediation pattern observed in the proposed model.

### 4.6 Group differences in AI dependency

To examine whether AI dependency differed across gender, academic year, and frequency of AI use, group difference analyses were conducted using independent-samples t-tests and one-way analysis of variance (ANOVA). The results indicated that AI dependency did not significantly differ by gender (*t*(274) = –1.053, *p* = 0.293). However, a significant difference was observed across academic years with a small effect size [*F*(3,272) = 2.858, *p* = 0.037, η^2^ = 0.031], and post-hoc comparisons further showed that senior students reported higher levels of AI dependency than junior students. Furthermore, AI dependency significantly varied across frequency of AI use [*F*(4,271) = 14.569, *p* < 0.001, η^2^ = 0.177]. The relatively larger effect size associated with AI usage frequency indicates that AI dependency differed substantially across levels of AI engagement.

The group difference analyses revealed that AI dependency varied according to academic year and AI usage frequency. However, these findings represent group differences rather than formal moderation effects, as the analyses did not test whether academic year or AI usage frequency altered the strength of the relationships among academic self-efficacy, teacher support, AI dependency, and learning burnout.

### 4.7 Robustness checks

To further examine the robustness of the findings, a series of additional analyses were conducted by including demographic variables as control variables in both the regression and mediation models. Specifically, gender, academic level, major category, frequency of AI use, and typical duration of AI use per session were controlled for in the analyses. The results showed that the inclusion of these control variables did not substantially alter the significance or magnitude of the main effects. Academic self-efficacy remained significantly and negatively associated with AI dependency (β = −0.424, *p* < 0.001), and teacher support continued to be negatively associated with AI dependency (β = −0.217, *p* < 0.001). In addition, AI dependency remained significantly and positively associated with learning burnout (β = 0.451, *p* < 0.001). Furthermore, the bootstrap confidence intervals for the indirect effects did not include zero, indicating that the mediating relationship remained statistically significant after controlling for demographic factors.

## 5 Discussion

### 5.1 Interpretation of key findings

This study explored the mediating role of AI dependency in the relationships between academic self-efficacy, teacher support, and learning burnout among university students in AI-assisted learning environments. The findings revealed that academic self-efficacy was negatively associated with AI dependency, indicating that students with stronger confidence in their learning abilities were less likely to rely excessively on AI tools. This finding is consistent with previous research showing that students with higher self-efficacy are more likely to engage in self-regulated learning and rely less on external cognitive supports (Li et al., 2024). Notably, some studies have also suggested that students with higher self-efficacy tend to show greater willingness in adopting AI-based learning technologies (Mohamud et al., 2026). These observations can be reconciled by distinguishing adaptive AI use and maladaptive AI dependency. Students with higher self-efficacy may be more likely to use AI as a learning aid while avoiding AI dependency. Building on previous studies that primarily examined the role of self-efficacy in academic achievement and motivation, this study further extends this perspective by demonstrating that self-efficacy also shapes students' AI-related behaviors, particularly by reducing AI dependency.

The results also showed that teacher support was negatively associated with AI dependency. This finding suggested that sufficient instructional guidance, emotional encouragement, and timely feedback from teachers could reduce students' AI dependency. This finding extends previous studies indicating that teacher support enhances students' engagement, motivation, and psychological wellbeing (Tao et al., 2022; Su et al., 2024). While previous research has primarily considered teacher support in traditional learning contexts, this study further demonstrates its importance in shaping students' AI-related learning behaviors.

In addition, AI dependency was positively related to learning burnout. Most previous empirical research has focused on the educational benefits of AI use, such as enhancing learning efficiency and providing personalized support (Kasneci et al., 2023). However, the potential adverse consequences of AI dependency remain relatively underexplored, particularly its relationship with learning burnout. While AI tools can facilitate learning as supportive resources, AI dependency may shift AI from a supportive resource to a substitute for students' own cognitive efforts. Over time, such dependency may reduce students' motivation and increase passive learning behaviors, ultimately intensifying learning burnout. This interpretation is consistent with Cognitive Offloading Theory and Conservation of Resources Theory, suggesting that excessive cognitive delegation to external technologies may lead to gradual depletion of psychological and cognitive resources (Risko and Gilbert, 2016; Halbesleben et al., 2014).

Furthermore, the mediating analysis demonstrated that AI dependency mediated the relationships between academic self-efficacy and learning burnout, as well as between teacher support and learning burnout. This finding indicates that both personal psychological factors and environmental support factors are associated with learning burnout through their effects on students' AI dependency behaviors. Previous research on student burnout has mainly examined psychological factors, such as self-efficacy and motivation, and contextual factors, such as social support (Salanova et al., 2010; Ye et al., 2021). This study extends previous research by identifying AI dependency as an emerging technology-related behavioral factor associated with students' learning burnout in AI-assisted learning contexts.

### 5.2 Theoretical contributions

This study contributes to the existing literature in several ways. First, it highlights the mediating role of AI dependency and broadens the literature on technology-related learning behaviors. Existing research has often examined how AI usage is related to learning outcomes (Abbas et al., 2024; Boubker, 2024; Wu and Yu, 2024). However, limited attention has been given to AI dependency as a distinct psychological and behavioral phenomenon and its potential implications for students' learning experiences and psychological outcomes. By positioning AI dependency as a potential resource-depletion process, the proposed model extends existing AI-use and dependency research by linking its antecedents, potential mechanism, and learning outcome within a unified framework. Specifically, it identifies academic self-efficacy and teacher support as individual and contextual antecedents of AI dependency and links AI dependency to learning burnout. From a Conservation of Resources perspective, these elements are integrated to explain how personal and contextual factors may shape AI dependency and, in turn, its association with learning burnout.

Second, this study extends the application of Social Cognitive Theory and Conservation of Resources Theory, by identifying AI dependency as a critical behavior influencing students' learning burnout. Although previous research has primarily emphasized the benefits of AI tools for improving learning efficiency and academic performance (Nurtayeva et al., 2023; Bećirović et al., 2025), the potential risks associated with AI dependency remains relatively underdeveloped. This study extends Social Cognitive Theory by showing that academic self-efficacy shapes not only internal self-regulation but also students' reliance on AI for managing cognitive demands, thereby incorporating AI dependency into the self-regulation framework. Furthermore, this study broadens the Conservation of Resources Theory perspective by identifying AI-assisted cognitive support as a novel cognitive resource with both preserving and depleting effects. Although AI tools may initially conserve cognitive resources, excessive dependency on AI may gradually undermine students' autonomous cognitive resources and contribute to learning burnout.

Third, by incorporating teacher support as an antecedent of AI dependency, this study extends the role of contextual factors in explaining students' AI dependency. Previous studies have primarily examined teacher support in relation to traditional outcomes such as academic achievement, student engagement, and learning motivation (An et al., 2022). In the context of AI-assisted learning, this study further demonstrates how supportive instructional environments shape students' AI dependency.

Finally, this study integrates Conservation of Resources Theory, Cognitive Offloading Theory and Social Cognitive Theory, to explain the psychological processes associated with learning burnout in AI-powered learning environments. Rather than attempting to capture all possible determinants of AI dependency, this study focuses on two theoretically meaningful independent variables representing individual capability beliefs and social learning environments. By simultaneously considering psychological factors (academic self-efficacy), contextual factors (teacher support), and behavioral factors (AI dependency), the study provides a more comprehensive account of students' learning experiences in the era of AI.

### 5.3 Practical implications

The findings of this study provide several practical implications for educators, higher education institutions and policymakers. First, educators should provide more effective academic and emotional support. Timely feedback, personalized guidance, and positive teacher-student interactions can help students develop stronger learning motivation and self-efficacy when completing academic tasks. Rather than completely restricting AI usage, educators should adopt meaningful integration of AI technologies, guiding students to use AI as a supportive learning aid.

Second, higher education institutions should carefully design structured learning environments that promote the appropriate use of AI technologies. In particular, AI literacy education should be incorporated into the curriculum systems. For example, relevant knowledge can be added to general education and professional courses. Moreover, institutions should place greater emphasis on cultivating students' self-regulated learning skills, enabling them to effectively manage their learning processes when using AI-powered tools.

Third, policymakers should establish clear guidelines for the appropriate use of AI tools. These guidelines should emphasize responsible AI use, academic integrity, and the prevention of over-dependency on AI. Policy efforts should also support the development of AI literacy standards that encourage students to critically evaluate and appropriately integrate AI tools into their learning processes.

### 5.4 Limitations and future research directions

However, this study has several limitations. First, this study adopted a cross-sectional research design to examine the relationships among academic self-efficacy, teacher support, AI dependency, and learning burnout. However, this method limits the ability to establish causal relationships among the variables and fails to capture their dynamic changes over time (Maier et al., 2023). In particular, although this study proposed that AI dependency contributes to learning burnout based on theoretical considerations, reciprocal relationships may also exist, such as the possibility that higher levels of learning burnout may lead students to develop greater AI dependency. Future research could employ longitudinal or experimental designs to better clarify the temporal sequence and potential reciprocal relationships among these variables, as well as to further validate the proposed model.

Second, the data were collected using self-report questionnaires in this study, which required participants to evaluate and report their own levels of academic self-efficacy, perceived teacher support, AI dependency, and learning burnout. Although this approach is widely used in educational and psychological research (Chang and Kuo, 2025; Kuo and Chang, 2025; Liu et al., 2025), it may still be influenced by social desirability bias and subjective perceptions, potentially leading to either overestimation or underestimation of the actual constructs (Podsakoff et al., 2003). In addition, although the adapted AI Dependency Scale demonstrated satisfactory reliability and construct validity, its content validity was not further assessed through expert review or pilot testing before the main survey. Future research should further validate the scale across diverse educational contexts and incorporate multiple data sources or marker-variable techniques to enhance measurement validity and reduce potential response bias.

Third, the sample was limited to undergraduate students from several comprehensive universities in Chengdu, China. The use of convenience sampling within a specific geographical and educational context may introduce sampling bias and restrict the external validity of the findings. AI dependency may vary across educational and cultural contexts because teaching practices, institutional AI policies, and teacher–student relationships may shape the availability and acceptability of AI-supported learning, while academic expectations and learner autonomy may influence students' AI dependency. Accordingly, the findings should be considered within the sampled Chinese undergraduate context and not generalized to other educational or cultural contexts. Future studies should include students from diverse regions, educational backgrounds, age groups, and academic levels using more representative sampling approaches to validate the applicability of the proposed model. In addition, CFA was used to establish the adequacy of the measurement structure before the PROCESS mediation analysis. However, PROCESS uses observed composite scores rather than latent variables. It does not simultaneously estimate the measurement and structural components or explicitly account for measurement error as latent-variable SEM does. Future research could employ latent-variable SEM with bootstrapped indirect effects and larger, more diverse samples to further validate the proposed mediation framework.

Finally, this study primarily focused on academic self-efficacy and teacher support as antecedent factors associated with AI dependency. Other educational and individual factors, such as self-regulated learning, intrinsic motivation, critical thinking, and academic engagement, were not fully incorporated into the research framework. These factors may also contribute to variations in students' AI dependency and learning outcomes. In addition, potential boundary conditions, including AI literacy, digital competence, frequency of AI usage, academic discipline, personality traits, and academic achievement, were not examined in this study. Although the group difference analyses showed that AI dependency varied across academic years and levels of AI usage frequency, these findings represent group differences rather than formal moderation effects and do not establish whether these characteristics alter the strength of the proposed relationships. Moreover, AI dependency may vary across educational systems due to differences in curriculum design, assessment practices, technology accessibility, and institutional policies regarding AI use. These factors may influence the strength of the associations among the variables and should be further investigated in future research. Future research could integrate additional antecedents, formally examine boundary conditions through moderation or moderated mediation analyses, and consider diverse educational contexts to develop a more comprehensive understanding of students' AI dependency and its relationships with learning outcomes in higher education.

## 6 Conclusion

As AI technologies are increasingly incorporated into higher education, university students' learning processes and academic behaviors are undergoing substantial changes. Accordingly, AI dependency has emerged as a critical behavioral factor that may significantly shape students' learning experiences and outcomes. This study developed a mediation model in which AI dependency functioned as an intermediary variable in the association between academic self-efficacy, teacher support, and learning burnout among university students. A quantitative cross-sectional research design was employed, with data collected from university students through an online structured questionnaire survey. Subsequent statistical analyses were performed to examine the reliability and validity of the measurement constructs and to test the proposed relationships among the variables. The results revealed that both academic self-efficacy and teacher support were negatively associated with learning burnout and negatively related to AI dependency, suggesting that students with stronger academic self-efficacy and higher levels of teacher support were less likely to rely on AI technologies in their learning process. In addition, AI dependency showed a significant positive association with learning burnout, suggesting that higher levels of AI dependency were associated with greater emotional exhaustion and reduced learning engagement. More importantly, AI dependency was identified as a significant mediating variable in the association between academic self-efficacy, teacher support, and learning burnout. This indicated that the effects of both psychological and contextual factors on learning burnout were transmitted through students' AI dependency. The findings highlighted that AI dependency played an important role in explaining students' learning burnout in AI-supported learning environments, functioning as a critical explanatory factor associated with the relationship between psychological and contextual factors and learning burnout. The findings provided practical implications for educators, higher education institutions, and policymakers, particularly in promoting responsible AI use and reducing learning burnout in AI-supported learning environments.

## Data Availability

The raw data supporting the conclusions of this article will be made available by the authors, without undue reservation.
